# Image Enhancement for Tracking the Translucent Larvae of *Drosophila melanogaster*

**DOI:** 10.1371/journal.pone.0015259

**Published:** 2010-12-30

**Authors:** Sukant Khurana, Wen-Ke Li, Nigel S. Atkinson

**Affiliations:** Section of Neurobiology and Institute for Neuroscience, The University of Texas at Austin, Austin, Texas, United States of America; University of Missouri, United States of America

## Abstract

*Drosophila melanogaster* larvae are model systems for studies of development, synaptic transmission, sensory physiology, locomotion, drug discovery, and learning and memory. A detailed behavioral understanding of larvae can advance all these fields of neuroscience. Automated tracking can expand fine-grained behavioral analysis, yet its full potential remains to be implemented for the larvae. All published methods are unable to track the larvae near high contrast objects, including the petri-dish edges encountered in many behavioral paradigms. To alleviate these issues, we enhanced the larval contrast to obtain complete tracks. Our method employed a dual approach of optical-contrast boosting and post-hoc image processing for contrast enhancement. We reared larvae on black food media to enhance their optical contrast through darkening of their digestive tracts. For image processing we performed Frame Averaging followed by Subtraction then Thresholding (FAST). This algorithm can remove all static objects from the movie, including petri-dish edges prior to processing by the image-tracking module. This dual approach for contrast enhancement also succeeded in overcoming fluctuations in illumination caused by the alternating current power source. Our tracking method yields complete tracks, including at the edges of the behavioral arena and is computationally fast, hence suitable for high-throughput fine-grained behavioral measurements.

## Introduction

Both the imago and larvae of *Drosophila melanogaster* have been classical tools for neuroscience and biology in general for over a century [1]. Larvae have been workhorses for many aspects of behavioral neuroscience, including sensory research [2]–[9] and learning and memory research [10]–[16]. Recently larvae have also been employed for drug discovery [17], [18]. As a model system, *Drosophila* larvae have many advantageous features for neuroscience research, including a plethora of molecular tools, rich and robust behavioral paradigms, an emerging electrophysiological/optophysiological toolkit, and the ease and economy with which they can be reared.

The ability to measure detailed larval behavior is vital for advancing larval neuroscience research. In many established behavioral paradigms, a human observer acts as the data collection device. Repeated human measurements can be time consuming and tedious, and human observations do not scale well. Human observations suffer from lack of temporal resolution. They are vulnerable to subjective bias of the experimenter and in the long run can be economically expensive. The temporal resolution of measurements recorded by a human observer is inherently limited to the speed at which the observer can count and take notes. This can be partially alleviated by using a camera to record the images for later analysis, but the workload for the experimenter can quickly pile up. Observations such as instantaneous position, speed, angular velocity, orientation strategies are beyond a human experimenter's ability to measure precisely. Scaling of behavioral measurements, needed for better statistical analysis and high throughput screening, is difficult with manual observation. Proper experimental setup and using multiple experimenters can partially resolve the issue of experimenter bias, but at times this can be economically prohibitive.

Detailed behavioral analysis can significantly benefit from the incorporation of automated tracking. Automated tracking cannot replace the intuition and intelligence of a human observer needed to establish a new behavior paradigm. What it can do, however, is to rapidly record large amount of detailed, precise data in previously established paradigms. This preserves the use of human intelligence and intuition for analysis instead of observation of behavior.

Automated tracking of the *Drosophila* larvae has proven to be difficult. This is due to the translucency of the larvae, which have low contrast against the background. All previously published methods cannot resolve larvae when they approach a high contrast static object such as a petri-dish edge. In effect, the image of the larvae merges with the object and the position of the larvae is lost [3]. The resulting incomplete tracks limit the type of analysis that can be performed. For example, the inability to obtaining complete tracks severely hinders the analysis of orientation strategy of the animal and the exploration of dynamic decision making by larvae.

In this study we resolve the inability to observe the larval behavior in detail by developing a new method that is capable of reliably tracking *Drosophila* larvae in standard larval assays. We were able to achieve this by enhancing the contrast of the larvae by feeding it black food dye and by employing a frame subtraction image processing method that allowed for much greater sensitivity than previous methods.

## Materials and Methods

### Larval behavior assay

0.8 ml of Black food dye (McCormick, www.mccormick.com, Universal product code 052100581873) was added to 50 ml of standard cornmeal/molasses/agar media containing early 3rd instar larvae for 6–12 hours before experiments. The larvae were extracted from the dyed media using density separation with 30% poly-ethylene-glycol (M.W. 1500) and kept in Ringer's solution until use in experiments [19], [12]. The Ringer's solution contained 128 mM NaCl, 4.7 mM KCl, 1.8 mM CaCl_2_, 0.9 mM Na_2_HPO_4_, and 0.37 mM KH_2_PO_4_ [20]. To study larval locomotion, larvae were placed onto the center of a 14 cm plastic petri dish containing 15 ml of 2% agar and allowed to crawl freely for 3.5 minutes. All movies were recorded for the entire duration of the test.

### Movie capture

A Unibrain Fire-I monochrome camera (Unibrain, www.unibrain.com) was used to capture movies. This camera is Instrumentation and Industrial Digital Camera (IIDC) standards compliant, and is capable of capturing at 30 frames per second at a resolution of 640 by 480 pixels. The camera was positioned at least 40 cm from the larval petri-dish. This prevented the camera from casting a shadow on the dish, which could influence larval behavior. The petri-dish was placed on top of a light table that provided uniform lighting to the dish from the bottom. The light table used four 12-watt compact fluorescent light (CFL) bulbs. Each light was positioned at 19 cm from the center of the illumination platform in a rectangle of 29 cm by 24 cm. Two acrylic glass diffusers were used, at 2 cm and 16 cm above the light bulbs respectively. The second diffuser was placed on top of a glass panel. The petri dish rested on the second acrylic diffuser. The movies were captured at 3.75 frames per second. The software used for recording the movies utilized the Image acquisition toolbox in Matlab (Mathworks, Natick, Massachusetts, USA; www.mathworks.com).

### Data analysis

For all descriptive statistics reported in results, we present mean ± standard deviation. All statistical significances were calculated using Student's t-test with p<0.01.

## Results

In this paper we explored two image-enhancement methods to improve the reliability of larval tracking. These were a physical contrast enhancement method and a post-hoc image processing method.

### Dye feeding enhances larval contrast

Larvae are optically translucent. This translucency produces low contrast against lit backgrounds, thus making tracking difficult. To alleviate this issue, we increased the contrast of the larvae by feeding the larvae food containing black food dye for 6–12 hours before each experiment. This period is sufficient for larvae to ingest the dye.

**Figure 1:**
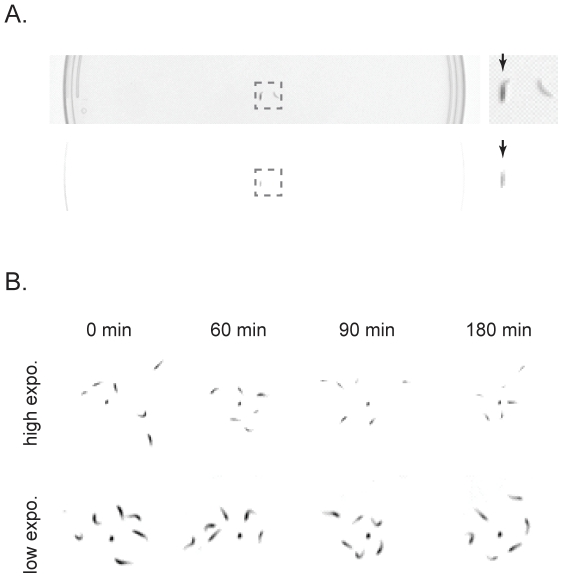
Feeding larvae black dyed food enhanced contrast. A. Dyed and control larvae in 15 cm plastic petri-dish under recording camera. Top: Dyed and control larvae under low exposure. Bottom: Same larvae under high exposure, where the petri-dish edge is barely visible. Arrows indicate dyed larva. B. Dye fed larvae were visible under high exposure for up to 3 hours after extraction.

Figure 1 shows the result of the enhanced contrast of larvae that is produced by dye feeding. We overexposed the image by increasing the exposure time. This reduced the visibility of lighter objects such as the petri-dish edges in the movie. Even when overexposed, the dye-fed larvae remained visible in the petri dish, while larvae without food dye were invisible (Figure 1a).

To assess if the contrast enhancement of larvae due to dye feeding persisted long enough for a behavioral experiment, we evaluated the perdurance of the dye. Over the period of collecting more than 3000 larval tracks, we observed that the dye fed larvae remained visible under conditions of overexposure for 2 to 4 hours after extraction from the food media. The contrast enhancement continued until the animals excreted the black food from their digestive tracts. Figure 1b illustrates an example in which larvae were visualized for 3 hours.

To determine whether the dye degraded the health of the larvae, we compared the number of pupae emerging from culture media bottles with and without dye. We compared of 40 bottles of each condition (mean ± SD pupae in dyed bottles = 117.3±27.2, control = 114.18±30.3). Using a two tailed t-test we found that the differences were not statistically significant (p-value = 0.63). In addition, we reared both the larvae and adult flies on food media containing dye for 3 generations. The viability of these flies was indistinguishable from flies reared on standard cornmeal media. In general, no change was observed in larval health, locomotion, sensory response or learning abilities (data not shown).

### Frame Averaging followed by Subtraction then Threshold (FAST) improved tracking

**Figure 2:**
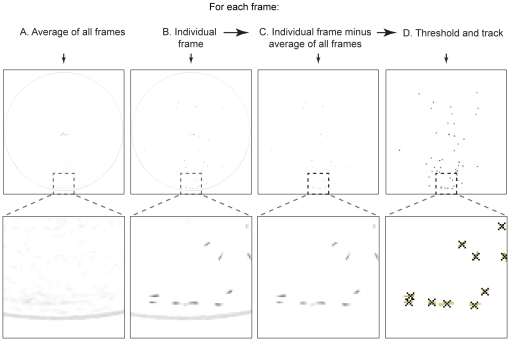
Larvae in the videos were tracked using the Frame Averaging followed by Subtraction then Thresholding method (FAST). We improved video tracking by subtracting individual frames from the average of all frames. The tracking algorithm is as follows: A. For each video, calculate an average of all the frame values. B. Obtain each frame in the video. C. Calculate difference between each frame and the average frame value. D. The result was then analyzed using a binary threshold process. For better visualization the larvae are represented as dark pixels on light background for C and D.

In order to maximize our ability to obtain complete larval tracks, we also pursued a post-hoc image processing method to reduce noise and remove static objects. We achieved this by using Frame Averaging followed by Subtraction then Thresholding (FAST), as illustrated in Figure 2. We first calculated the average value of all the frames. Then, for each individual frame we calculated the value of the average frame minus the individual frame. Using this algorithm, we were able to remove all static objects from the movie including the petri-dish edges. We then applied the binary threshold method to the resulting frames, and tracked all moving objects that passed the threshold. The binary threshold method first transforms a grayscale image into a binary image by applying a threshold to each pixel of the image. The resulting pixel clusters that passed the threshold are labeled as larvae. The center of each cluster was recorded as the position of the larva. With FAST, we could use a very low threshold without interference from static objects since they were removed by frame subtraction.

The binary thresholding and tracking is a standard algorithm available in common open source toolkits such as OpenCV (opencv.willowgarage.com). It has been previously employed by other labs [21]. The implementation of the algorithm used in this paper are standard procedures in Matlab. We used the command “im2bw” for binary thresholding of the image. We then employed the command “bwlabel” to label the connected white pixel clusters. We employed the command “regionprops” to find the centers and sizes of these clusters. The clusters are pruned based on a minimal and maximal threshold of 2 and 100, respectively. The tracks were generated by comparing these centers with centers from the previous frame. The closest centers were connected as tracks, and appended to existing tracks if the distance between two centers is less than 10 pixels (approximately 3 mm) [21].

**Figure 3:**
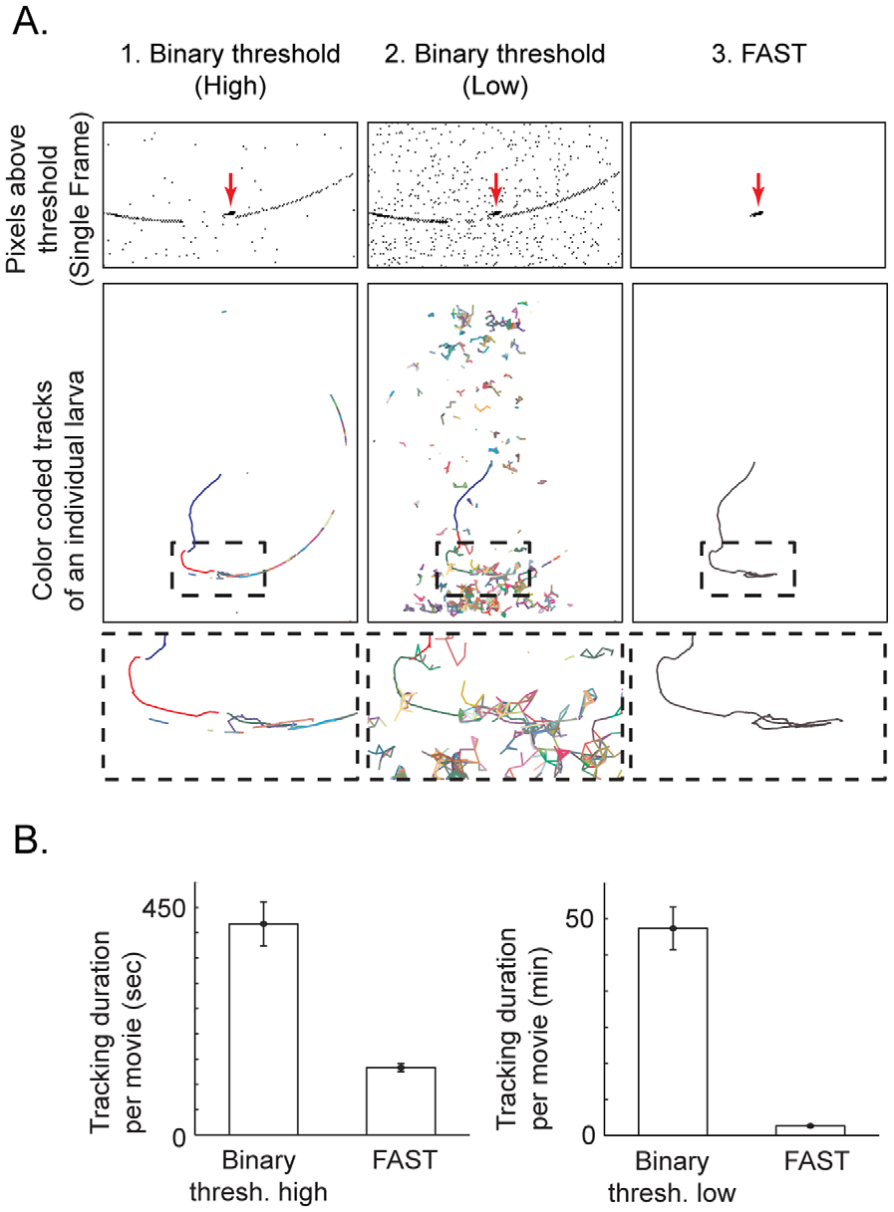
FAST allowed increased sensitivity for tracking. A. FAST was able to obtain the complete track of the larva. 1. With the binary threshold method, applying a high threshold (0.09) resulted in incomplete tracks as well as noise. 2. On the other hand, applying a low threshold (0.073) resulted in noise levels too high to reliably generate complete larval tracks. 3. In contrast, using FAST with a very low threshold (0.028) produced little noise and allowed for reliable generation of complete tracks. Top row: magnified view of a single movie frame showing pixels above threshold for each method. For visualization these pixels are represented as black pixels on white background. FAST was able to isolate the larva while eliminating other noise. Arrows indicate the larva. Middle row: tracks generated using each method. Each track segment of the larva is represented by a different color. Bottom row: magnified view of middle row. B. FAST was at least 3 times faster per movie than using binary threshold method with high threshold and 20 times faster than using low threshold (n = 5).

Compared to simply applying a binary threshold to the raw movie images, FAST allowed for much more reliable detection of the larvae (Figure 3). When using a high threshold (0.09 in this example) for the movie images, the larvae tracks are broken due to lack of sensitivity (Figure 3a). When that threshold is lowered (0.073 effective in this example), there was excessive noise for reliable tracking (Figure 3a). However, when the FAST was used, the threshold can be much lower (0.028 for all movies) with very little noise, thus allowing reliable tracking of larvae (Figure 3a). The ability to use a fixed threshold for tracking all movies recorded in various conditions meant there was no manual intervention necessary, speeding up the processing of movies. As an added benefit, using FAST resulted in significantly faster processing time per movie (Figure 3b), due to reduced noise (Figure 3a, top row). The processing time using FAST was on average 133±8 seconds (n = 5), compared to 417±43 seconds (n = 5) for high binary threshold alone, and 2867±294 seconds (n = 5) for low binary threshold alone. The p-values were found to be less that 0.01 for both the differences between FAST and high binary threshold and between FAST and low binary threshold. The reduced computation time required for tracking was due to the decreased number of objects visible in the movie after frame subtraction. The processing time includes both image processing and tracking. Thus, FAST allowed us to quickly and reliably track dyed larvae, which is crucial for high throughput screening of larval responses.

### Dye feeding and average frame subtractions are both necessary for reliable larval tracking

**Figure 4:**
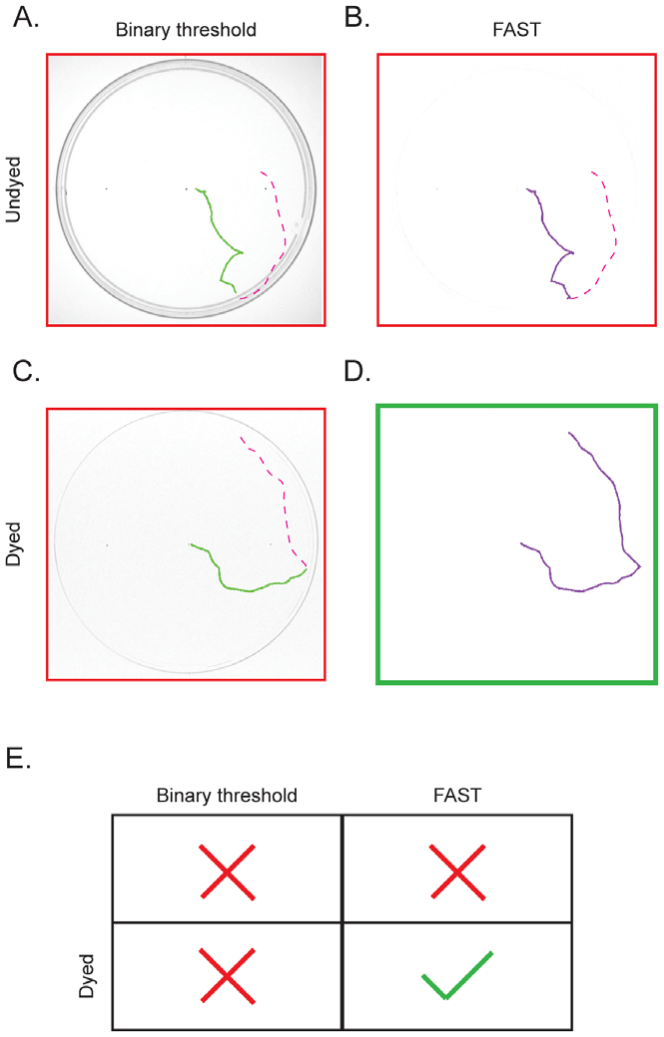
Dye-fed larvae and FAST are both necessary for reliable larval tracking. A. Without dye and FAST, the larva could not be followed once it encounters the petri-dish edge. B. FAST without dye also cannot reliably follow the larva when it encounters the edge. C. Similarly, using dyed larva without FAST results in a failure to follow the larva near the edge. D. Only when both dyed larva and FAST were used in conjunction can the larvae be reliably tracked near the edge. The same movie was used between panels A., B. and C., D. Red dashed lines indicate the untracked portion of the larval track. For better illustration C and D are show as dark tracks on white background. We concluded that both the dye-fed larvae and FAST are necessary for reliable larval tracking (E).

While FAST proved to be much more sensitive for tracking the larvae in the petri-dish, it was still necessary to enhance the contrast of the larvae with black food dye in order to reliably generate complete tracks (Figure 4). This is due to the fact that the exposure value at which the undyed larvae could be resolved in the movie also resulted in very visible dish edges, such that when larvae merged with the dish edge they became indistinguishable from the edge. Under such circumstances, unless one employs a very high resolution imaging, FAST alone would subtract away the larvae along with the edge, resulting in incomplete tracks. This is apparent in Figure 4b, where the larval track ends abruptly at the petri-dish edge even using FAST. The binary threshold method performed even worse, terminating the track before the edge (Figure 4a). As discussed previously, even with dye feeding, the binary threshold method is still incapable of reliably tracking the larvae when it hits the edge (Figure 4c). Only when we employed both dye feeding and FAST approach together could the larvae be tracked after it had run into the edge (Figure 4d).

### Extracting locomotion parameters from tracks

**Figure 5:**
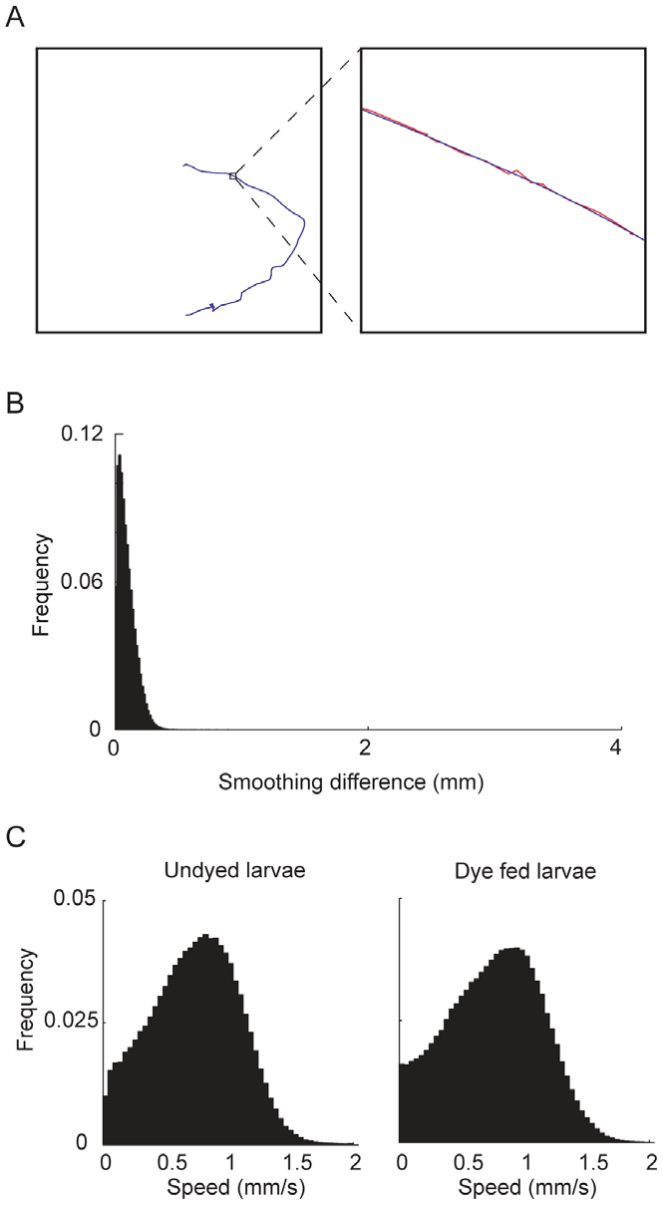
Extraction of locomotion parameters from tracks. A. We employed a Gaussian smoothing algorithm to the tracks. Red: original track, blue: smoothed track. B. The Gaussian smoothing did not introduce major differences from original tracks. Greater than 90% of differences were less than 200 µm. C. The speed distributions were not noticeably different between undyed and dye fed larvae (100 larvae for each distribution). To generate the distribution we calculated the instantaneous speed for each point on the smoothed track and plotted the distribution of all the speeds.

The tracks allowed us to extract detailed locomotion parameters (Figure 5). In order to reduce the possible wobble caused by image noise, we first applied Gaussian smoothing [22] to the original tracks (Figure 5a). The Gaussian smoothing did not introduce major differences from the original track, with the majority of differences below 200 µm, which is 1/20th of the length of a normal 3rd instar larva (Figure 5b). We then calculated the instantaneous speed of the larvae at each point of the smoothed track. In order to ensure that dye feeding did not significantly alter larval locomotion, we plotted the instantaneous speed distribution of the dye fed larvae and compared it to that of undyed larvae (Figure 5c). There was almost no difference (0.02 mm/s) between the speed of dyed and undyed larvae. (mean dyed speed = 0.76 mm/s, 100 larvae; mean undyed speed = 0.74 mm/s, 100 larvae). Our method is designed to track single animals as we have not tried to resolve the issue of track intersection. To explore the number of animals that can be used in a 14 cm petri-dish with significant track durations, we measured the average duration of tracks for different population sizes. We obtained average (mean ± SD) track duration of 165±53 s (n = 2570 tracks), 160±80 s (n = 114 tracks), 154±99 s (n = 1601 tracks), 135±72 (n = 470 tracks) for 1, 5, 10 and 20 animals respectively.

In summary, with our ability to reliably track the larvae through dye feeding and FAST, we developed a method to examine larval behavior in much greater detail than previously possible.

## Discussion

In this study we have developed an improved method of tracking larvae that resolves their position, even at the edges of behavioral arena. We employed a dual approach of enhancing larval contrast by dye feeding and static objects removal using Frame Averaging followed by Subtraction then Thresholding (FAST). Using this approach, we are able to generate complete larval tracks.

Given the number of *Drosophila* behavioral studies, it is surprising that larval tracking has not become well established. The availability of various particle trackers for use in diverse situations would seem to render our effort to develop a method to track larvae redundant. Unfortunately, for many old open source “bug trackers” [23]–[25], either the software or the hardware components are not readily available. Furthermore, the commercially available packages are expensive and poorly adaptable for tracking translucent animals. A previous image analysis system developed by Ramazani *et al*. [26] is very effective at analyzing the activity of adult flies by counting the number of pixels present after frame subtraction. However, it was not designed for tracking the path taken by animals. The method that we have presented here enables us to analyze the detailed movement of individual larvae. Our study does not deal with tracking populations and hence is not designed to resolve track intersection. Many studies in other systems, which employ either machine learning or deterministic filters have been used successfully to track populations of animals [27]–[29]. As larvae become more commonly used in neuroscience research, the use of these techniques for resolving multiple animals is likely to become very important for tracking larvae.

Apart from being able to completely track larvae, our approach has several other advantages, such as economy, flexibility of both hardware and software, resistance to light level fluctuations, and open source provision from our end. Furthermore, because the software is open source, it can be modified and improved by other investigators.

The simple, inexpensive hardware and software in our solution means that the overall cost of the system is very low. Our method costs at least ten fold less than commercially available systems like Ethovision (Noldus Information Techonology, Wageningen, Netherlands; http://www.noldus.com/). This economic advantage allows for more data acquisition rigs, facilitating bigger scaling up. We plan to provide our tracking software as open source.

The Instrumental and Industrial Digital Camera (IIDC) standard compliant camera we use has two major advantages: flexibility and control. Various software and software libraries are available to record from IIDC cameras, including open source solutions such as Coriander (damien.douxchamps.net/ieee1394/coriander/) and the 1394-based DC Control library (sourceforge.net/projects/libdc1394). This flexibility means that the hardware is not locked to any single proprietary software solution. IIDC standard compliance also gives one the flexibility to upgrade the hardware and/or software easily without changing any other component. Finally, IIDC standard gave us complete software control over the exposure time and other parameters of the camera. It also allows one to record uncompressed movies, which simplifies image analysis. Camera systems such as digital video (DV) system, which is commonly used in handheld movie cameras, can only send compressed output to the computer. This results in degraded image quality.

When designing the software we had multiple options for implementation, such as C/C++, Java, and higher level languages, such as Matlab. Despite the fact that Matlab is not open source and requires a proprietary run-time package, we chose to use it due to its extensive built-in tools including computer vision algorithms, and its ease of programming and prototyping. In addition, data structure manipulation in Matlab is significantly easier than in lower level languages such as C/C++. Matlab also provides tools to enable its data structure to be easily read by other lower level languages, thus making the transition to other languages very easy, should the need arise.

The combination of the dye feeding and FAST method significantly reduced problems arising from light level fluctuations. The light level fluctuations observed in the movie recordings are due to the mismatch in the capture frequency of the camera and the light intensity oscillation frequency of the light table. Due to the alternating current nature of the power source for the light bulbs, the light output oscillated at twice the input frequency of the power source (60 Hz). In our setup the camera's internal timing mechanism captured a frame once every 1/30th of a second, regardless of the output frame rate. In reality the input frequency of the power source is never exactly 60 Hz, nor is the frame rate of the camera exactly 30 frames per second. These slight errors in timing introduce a mismatch in frequencies. This mismatch results in a slight phase shift in the light level captured at each frame. Over the course of many frames, this resulted in a light level fluctuation in the movie. This fluctuation in illumination might cause significant fluctuations in contrast of the larvae. Thus, to maximize the probability of obtaining complete tracks, the threshold for binary thresholding should be set very low so that even if the larvae is only slightly different from the background it will be detected. However, such a low threshold resulted in a corresponding increase in noise from static objects in the frame. We were able alleviate this issue using FAST. This allowed us to set the threshold of detection to be much lower than that in the binary threshold method, without much interference from noise. An additional method to reduce the impact of a highly fluctuating light source is to scale each frame (before subtracting) by computing the mean intensity over the entire field and normalize the frame to the mean of means. We did not need to use this scaling in our study but this approach can provide additional benefits over FAST in cases of severe light fluctuation.

The method we have described in this study allowed us to reliably observe and track the behavior of the larvae throughout the entire duration of standard larval behavioral assays for olfaction [2], [3], gustation [9], phototaxis [8], learning, and memory [12]. Apart from generating complete tracks we are able to track movies many times faster than binary thresholding, a feature critical for high throughput screenings. Our approach of feeding dye using dye-fed larvae and FAST allows for more subtle study of larval behavior.
